# YanHuang Paternal Genomic Resource Suggests A Weakly-differentiated Multi-source Admixture in the Formation of Han Founding Ancestral Lineages

**DOI:** 10.1093/gpbjnl/qzaf049

**Published:** 2025-06-04

**Authors:** Zhiyong Wang, Kaijun Liu, Haibing Yuan, Shuhan Duan, Yunhui Liu, Lintao Luo, Xiucheng Jiang, Shijia Chen, Lanhai Wei, Renkuan Tang, Liping Hu, Jing Chen, Xiangping Li, Qingxin Yang, Yuntao Sun, Qiuxia Sun, Yuguo Huang, Haoran Su, Jie Zhong, Hongbing Yao, Libing Yun, Jianbo Li, Junbao Yang, Yan Cai, Hong Deng, Jiangwei Yan, Bofeng Zhu, 10K_CPGDP Consortium, Guanglin He, Chao Liu, Mengge Wang, Renkuan Tang, Libing Yun, Junbao Yang, Chuan-Chao Wang, Jiangwei Yan, Bofeng Zhu, Liping Hu, Shengjie Nie, Hongbing Yao, Kun Zhou, Shengjie Nie, Chao Liu, Mengge Wang, Guanglin He

**Affiliations:** Department of Oto-Rhino-Laryngology & Institute of Rare Diseases, West China Hospital of Sichuan University, Sichuan University, Chengdu 610000, China; Center for Archaeological Science, Sichuan University, Chengdu 610000, China; Department of Forensic Medicine, College of Basic Medicine, Chongqing Medical University, Chongqing 400331, China; School of International Tourism and Culture, Guizhou Normal University, Guiyang 550025, China; MoFang Human Genome Research Institute, Tianfu Software Park, Chengdu 610042, China; Center for Archaeological Science, Sichuan University, Chengdu 610000, China; Department of Oto-Rhino-Laryngology & Institute of Rare Diseases, West China Hospital of Sichuan University, Sichuan University, Chengdu 610000, China; School of Basic Medical Sciences, North Sichuan Medical University, Nanchong 637100, China; Department of Oto-Rhino-Laryngology & Institute of Rare Diseases, West China Hospital of Sichuan University, Sichuan University, Chengdu 610000, China; Department of Forensic Medicine, College of Basic Medicine, Chongqing Medical University, Chongqing 400331, China; Department of Oto-Rhino-Laryngology & Institute of Rare Diseases, West China Hospital of Sichuan University, Sichuan University, Chengdu 610000, China; Department of Forensic Medicine, College of Basic Medicine, Chongqing Medical University, Chongqing 400331, China; Department of Oto-Rhino-Laryngology & Institute of Rare Diseases, West China Hospital of Sichuan University, Sichuan University, Chengdu 610000, China; School of Basic Medical Sciences, North Sichuan Medical University, Nanchong 637100, China; Department of Oto-Rhino-Laryngology & Institute of Rare Diseases, West China Hospital of Sichuan University, Sichuan University, Chengdu 610000, China; Department of Forensic Medicine, College of Basic Medicine, Chongqing Medical University, Chongqing 400331, China; School of Ethnology and Anthropology, Institute of Humanities and Human Sciences, Inner Mongolia Normal University, Hohhot 010022, China; Department of Forensic Medicine, College of Basic Medicine, Chongqing Medical University, Chongqing 400331, China; School of Forensic Medicine, Kunming Medical University, Kunming 650500, China; Department of Oto-Rhino-Laryngology & Institute of Rare Diseases, West China Hospital of Sichuan University, Sichuan University, Chengdu 610000, China; School of Forensic Medicine, Shanxi Medical University, Taiyuan 030000, China; Department of Oto-Rhino-Laryngology & Institute of Rare Diseases, West China Hospital of Sichuan University, Sichuan University, Chengdu 610000, China; School of Forensic Medicine, Kunming Medical University, Kunming 650500, China; Department of Oto-Rhino-Laryngology & Institute of Rare Diseases, West China Hospital of Sichuan University, Sichuan University, Chengdu 610000, China; School of Forensic Medicine, Kunming Medical University, Kunming 650500, China; Department of Oto-Rhino-Laryngology & Institute of Rare Diseases, West China Hospital of Sichuan University, Sichuan University, Chengdu 610000, China; Institute of Forensic Medicine, West China School of Basic Medical Sciences & Forensic Medicine, Sichuan University, Chengdu 610041, China; Department of Oto-Rhino-Laryngology & Institute of Rare Diseases, West China Hospital of Sichuan University, Sichuan University, Chengdu 610000, China; Department of Forensic Medicine, College of Basic Medicine, Chongqing Medical University, Chongqing 400331, China; Department of Oto-Rhino-Laryngology & Institute of Rare Diseases, West China Hospital of Sichuan University, Sichuan University, Chengdu 610000, China; Department of Oto-Rhino-Laryngology & Institute of Rare Diseases, West China Hospital of Sichuan University, Sichuan University, Chengdu 610000, China; Department of Clinical Laboratory, North Sichuan Medical College and Center for Genetics and Prenatal Diagnosis, Affiliated Hospital of North Sichuan Medical College, Nanchong 637007, China; Department of Oto-Rhino-Laryngology & Institute of Rare Diseases, West China Hospital of Sichuan University, Sichuan University, Chengdu 610000, China; Belt and Road Research Center for Forensic Molecular Anthropology, Gansu University of Political Science and Law, Lanzhou 730000, China; Institute of Forensic Medicine, West China School of Basic Medical Sciences & Forensic Medicine, Sichuan University, Chengdu 610041, China; Department of Forensic Medicine, College of Basic Medicine, Chongqing Medical University, Chongqing 400331, China; Institute of Basic Medicine and Forensic Medicine, North Sichuan Medical College and Center for Genetics and Prenatal Diagnosis, Affiliated Hospital of North Sichuan Medical College, Nanchong 637007, China; Department of Clinical Laboratory, North Sichuan Medical College and Center for Genetics and Prenatal Diagnosis, Affiliated Hospital of North Sichuan Medical College, Nanchong 637007, China; School of Forensic Medicine, Kunming Medical University, Kunming 650500, China; School of Forensic Medicine, Shanxi Medical University, Taiyuan 030000, China; Guangzhou Key Laboratory of Forensic Multi-Omics for Precision Identification, School of Forensic Medicine, Southern Medical University, Guangzhou 510515, China; Microbiome Medicine Center, Department of Laboratory Medicine, Zhujiang Hospital, Southern Medical University, Guangzhou 510515, China; MoFang Human Genome Research Institute, Tianfu Software Park, Chengdu 610042, China; School of Forensic Medicine, Kunming Medical University, Kunming 650500, China; Guangzhou Key Laboratory of Forensic Multi-Omics for Precision Identification, School of Forensic Medicine, Southern Medical University, Guangzhou 510515, China; Anti-Drug Technology Center of Guangdong Province, Guangzhou 510230, China; Guangzhou Forensic Science Institute, Guangzhou 510055, China; Department of Oto-Rhino-Laryngology & Institute of Rare Diseases, West China Hospital of Sichuan University, Sichuan University, Chengdu 610000, China; Center for Archaeological Science, Sichuan University, Chengdu 610000, China; Department of Forensic Medicine, College of Basic Medicine, Chongqing Medical University, Chongqing 400331, China; Guangzhou Forensic Science Institute, Guangzhou 510055, China; Faculty of Forensic Medicine, Zhongshan School of Medicine, Sun Yat-sen University, Guangzhou 510275, China; Department of Oto-Rhino-Laryngology & Institute of Rare Diseases, West China Hospital of Sichuan University, Sichuan University, Chengdu 610000, China; Center for Archaeological Science, Sichuan University, Chengdu 610000, China

**Keywords:** YanHuang cohort, Y-chromosome lineage, Han Chinese, Population structure, Evolution history

## Abstract

The revolution in large-scale human genomics and advancements in statistical methods have profoundly refined our understanding of genetic diversity and structure within human populations. Y-chromosome variations, with their distinct evolutionary characteristics, play crucial roles in reconstructing the origins and interactions of ancient East Asian paternal lineages. We launched the YanHuang cohort employing a high-resolution capture sequencing panel to explore the evolutionary trajectory of Han Chinese, one of the world’s largest ethnic groups. We generated paternal genomic data for 5020 Han Chinese individuals across 29 Chinese administrative regions. We observed that multiple founding paternal lineages originating from ancient western Eurasia, Siberia, and East Asia contributed significantly to the Han Chinese gene pool. We identified fine-scale paternal genetic structures shaped by interactions among ancient populations and geographic barriers like the Qinling-Huaihe line and the Nanling Mountains. These structures reflect both isolation-enhanced and admixture-driven genetic differentiation, underscoring the complexity of Han Chinese genomic diversity. We observed a strong correlation between the frequency of multiple founding lineages and subsistence-related ancestral sources, including western pastoralists, Holocene Mongolian Plateau populations, and ancient East Asians. This relationship highlights the impact of ancient migrations and admixture on Chinese paternal genomic diversity. We introduced the Weakly-Differentiated Multi-Source Admixture model to clarify the intricate interactions among multiple ancestral sources influencing the Han Chinese paternal landscape. This study provides a comprehensive uniparental genomic resource from the YanHuang cohort, proposes a novel admixture model, and delineates the complex genomic landscape shaped by ancient herders, hunter-gatherers, and farmers integral to Han Chinese ancestry.

## Graphical abstract

**Figure:**
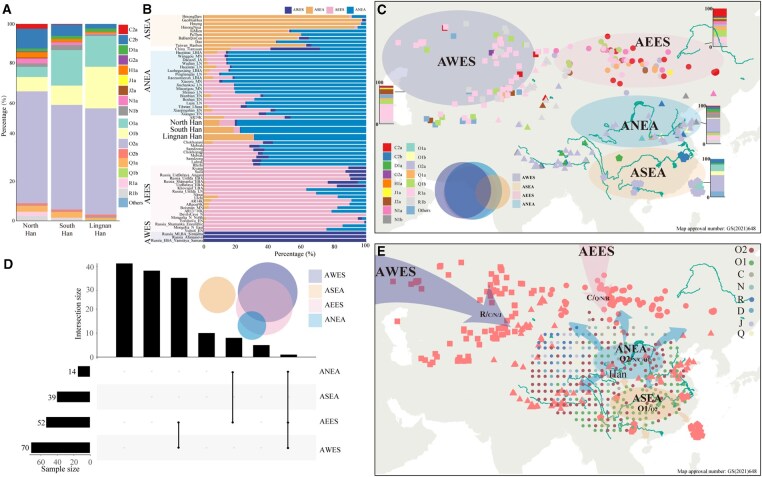

## Introduction

East Asia, positioned at the nexus connecting America and the Pacific Islands, exhibits significant ethnolinguistic diversity and is pivotal for studying human evolution [1]. Decades of genetic research have elucidated the origins, formation, and divergence of modern East Asians [2,3]. The initial peopling of East Asia, occurring tens of thousands of years ago, followed two major migration routes — northern and southern — establishing the foundational gene pool of East Asian ancestors [4]. The genetic variations in present-day East Asians are influenced by factors such as population admixture, social-cultural practices, and geographic isolation, indicating their different and complex population histories [5]. Advances in ancient DNA research have further refined our understanding of ancient East Asians’ evolutionary history [6,7]. Genetic turnover and differentiation have been demonstrated through ancient DNA analysis. Furthermore, genetic continuity, along with complex population interactions and key migration events, has been observed [8–10]. Mao et al. identified Tianyuan/Yana-related Paleolithic lineages that contributed to the gene pool of Siberians and northern East Asians prior to the Last Glacial Maximum (LGM) and reported genetic turnover between pre-LGM AR33K and LGM AR19K, as well as genetic continuity between post-LGM populations in the Amur region [8]. Concurrently, Ning et al. analyzed ancient DNA from northern China, uncovering evidence of population interactions shaped by migration and varied subsistence strategies [11]. Yang et al. identified a genetic stratification emerging in the early Neolithic, with ancient northern East Asians (ANEA), such as the Shandong Xiaogao and Xiaojingshan individuals, genetically distinct from ancient southern East Asians (ASEA), represented by the Fujian Qihe individuals [12]. Wang et al. further documented persistent southward gene flow from the Yellow River Basin (YRB), which influenced Neolithic through historical populations in Fujian and Guangxi [9]. Studies by McColl et al. traced additional southward dispersals from South China into Southeast Asia and Oceania, mirroring prehistoric population expansions and migrations [13]. Moreover, ancient western Eurasian Steppe (AWES)-related populations, including Yamnaya- and Afanasievo-affiliated lineages and later herders from Central Asia, contributed to the genomic landscape of ancient Eastern Eurasian Steppe (AEES) groups, which also possessed dominant Neolithic ancestry from the Mongolian Plateau and Amur River Basin [8,14]. Overall, these complex differentiation and admixture events among spatiotemporally diverse ancient Eurasian populations reshaped the genetic landscape of eastern Eurasians, aligning with the spread of diverse subsistence strategies [10]. However, the extent to which autosomal-derived ancient demographic events have influenced the uniparental gene pool of modern East Asians and the fine-scale uniparental genetic architecture of Han Chinese remains to be comprehensively investigated.

The Han Chinese, one of the world’s largest ethnic groups distributed across diverse East Asian regions, have been the focus of genetic, archaeological, and linguistic studies for over two decades [15–17]. These studies link the origins of Sino-Tibetan (ST) languages to Neolithic millet farmers in the upper-middle YRB and associate the dispersal of ST languages with the farming-language dispersal hypothesis [10,18]. These early millet agriculturalists made significant contributions to the genetic makeup of present-day Han Chinese populations [10]. Historical dispersal patterns and interactions with surrounding populations underscore the complex genetic history of Han Chinese [19]. Xu et al. introduced the “snowball theory” to explain the formation of the Han ethnic group, suggesting that Han Chinese expanded by assimilating cultural and genetic elements from neighboring groups [20]. Using uniparental markers, Wen et al. demonstrated the southward demic diffusion of the Han, highlighting a sex-biased admixture that differentiated genetic contributions between northern and southern Han along paternal and maternal lineages [21]. The phylogeographic distribution of Y-chromosome lineages revealed a varied and intricate genetic composition within Han Chinese, featuring diverse paternal lineages [4,22,23]. Concurrently, autosomal DNA studies have identified different ancestral components among geographically different Han Chinese populations [24–28]. However, limitations in sample size and a scarcity of ancient DNA data for East Asia have left significant gaps in understanding the genetic history and relationships between Han Chinese and other ancient East Asian populations. Moreover, the extent to which and how past populations with different subsistence strategies, such as indigenous farmers and neighboring pastoralists, have contributed to the paternal genetic makeup of Han Chinese remains uncertain.

Previous studies have employed various genetic markers to elucidate the complex population structure of Han Chinese individuals and their genetic interactions with ethnolinguistically and geographically diverse populations [3,29]. Early genome-wide genotyping data revealed north-to-south and east-to-west genetic divergence within the Han Chinese population, which is consistent with an isolation-by-distance model in which genetic relatedness diminishes with increasing geographic distance [30]. Recent whole-genome sequencing data, facilitated by advances in high-throughput sequencing, have deepened our understanding of the population stratification of Han Chinese individuals, reflecting the varied demographic histories across East Asia [5,31–33]. Unlike nuclear autosomal genome data, uniparentally inherited markers offer unique evolutionary insights into the sex-specific genetic landscape because of their non-recombining nature and sex-specific inheritance patterns [34,35]. Earlier genetic analyses using limited markers revealed a north-to-south gradient in the paternal and maternal genetic structures of Han Chinese individuals [21,22,36,37]. Comprehensive sampling by Li et al. highlighted fine-scale matrilineal genetic divergences linked to river barriers, emphasizing the profound impact of agricultural technological innovations on matrilineal genetic variations [38]. The paternal genetic architecture of Han Chinese has been extensively studied; however, the limited number of Y-chromosome single-nucleotide polymorphisms (Y-SNPs) employed and sampling biases have constrained our comprehension of the fine-scale paternal population structure and its key influencing factors [21,22,39]. Additionally, residence models such as patrilocality and matrilocality play distinct roles in the diversity and variation spectrum of the human Y-chromosome, further complicating the genetic landscape. This ongoing research underscores the need for more refined genetic analyses to fully understand the complex genetic foundation of Han Chinese individuals.

**Figure 1:**
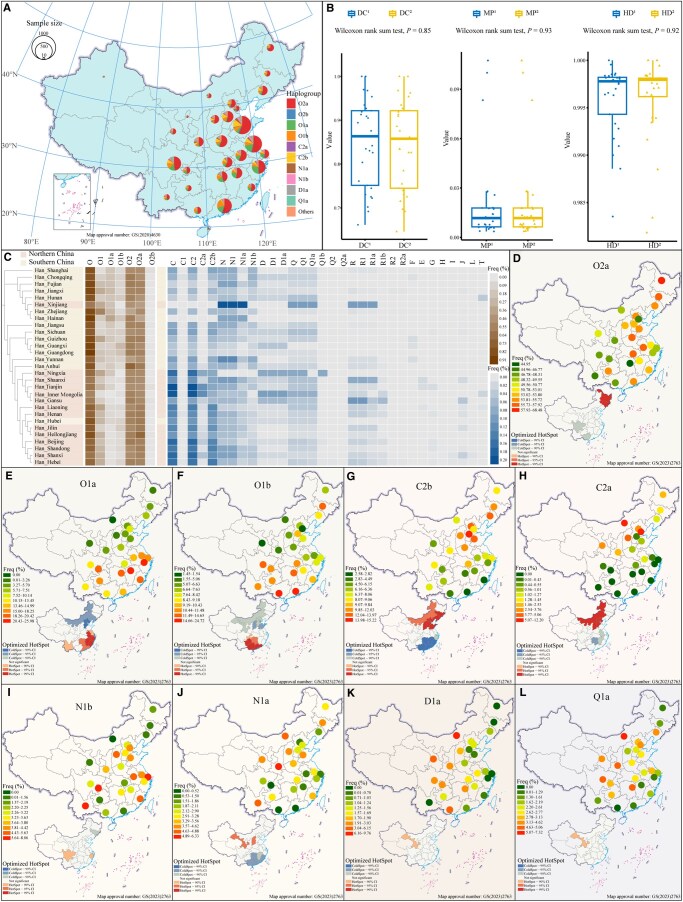
Geographical location of newly collected samples and haplogroup frequency distribution at the third level **A**. Geographical distribution of YanHuang cohort samples from 29 provincial-level administrative regions in China. Circle positions indicate sampling regions, with circle size proportional to sample size. Colors within circles indicate the proportions of different haplogroups. **B**. Box plots of DC, MP, and HD based on haplogroups and haplotypes among Han Chinese individuals. Superscripts 1 and 2 denote haplotype and haplogroup, respectively. **C**. Heatmap of the third-level haplogroup frequency spectrum among the Han Chinese populations. Tree topology is based on F_ST_ calculated from the third-level haplogroup frequency matrix. **D**.–**L**. Geographical distribution heatmaps of O2a (D), O1a (E), O1b (F), C2b (G), C2a (H), N1b (I), N1a (J), D1a (K), and Q1a (L) haplogroups at the third level among Han Chinese individuals. The upper map shows haplogroup frequencies, with colors representing frequency levels. The lower map shows spatial autocorrelation results, indicating HotSpot (high-value spatial clustering) and ColdSpot (low-value spatial clustering) regions, with CIs color-coded. Data from Xinjiang (10), Ningxia (12), and Hainan (11) were excluded due to their small sample sizes. DC, discrimination capacity; MP, match probability; HD, haplotype diversity; Freq, frequency; CI, confidence interval.

To further delineate the fine-scale paternal genetic structure and evolutionary history of Han Chinese individuals and investigate their formation mechanisms, we conducted the pilot work of the YanHuang cohort project, examining uniparental genomic resources from 5020 unrelated Han Chinese individuals across 29 provincial-level administrative regions in China (**Figure 1**A), utilizing the YHseqY3000 panel [40]. This panel was developed based on an extensive uniparental genomic database and the highest-resolution phylogenetic tree constructed as part of the 10K Chinese People Genomic Diversity Project (10K_CPGDP) [3]. This study has thus far provided one of the most extensive and comprehensive analyses of Y-chromosomal genetic variations and haplogroup frequency spectra (HFS) within the Han Chinese population. We investigated the fine-scale paternal genetic structure of the Han Chinese population and the possible factors influencing this structure, including cultural elements, river barriers, and mountainous divisions. Additionally, we analyzed the impact of diverse founding lineages shaped by extensive admixture events from historically isolated or differentiated sources on the Han Chinese gene pool since the Holocene. We proposed the Weakly-Differentiated Multi-Source Admixture (WDMSA) model, suggesting that multiple sources, including ancient western Eurasian herders, Siberian hunter-gatherers, and East Asian millet and rice farmers, have played roles in the formation of the paternal gene pool of Han Chinese. Overall, our work offers a comprehensive view of the paternal genetic history of Han Chinese, introducing a novel genetic WDMSA model that illuminates their evolutionary processes. We highlight two critical evolutionary forces — isolation-enhanced and admixture-introduced genetic differentiation — that have significantly shaped the paternal genetic landscape of the Han Chinese population.

## Results

### Paternal genetic diversity of the Han Chinese population in the YanHuang cohort

The YanHuang cohort was established to document the complete spectrum of paternal genetic diversity across geographically distinct Han populations. This initiative resulted in 5020 targeted Y-chromosome sequences, incorporating 2999 phylogenetically informative SNPs from the YHseqY3000 panel across 29 provincial-level administrative regions (Figure 1A; Table S1). After quality control, we identified 1899 unique haplotypes and 1766 terminal haplogroups, which were classified via our specially developed forensic phylogenetic tree (Table S2). The haplotype and haplogroup diversity values were strong and consistent, ranging from 0.9835 to 1.0000 and from 0.9818 to 1.0000, respectively (Figure 1B; Table S3). The discrimination capacity (DC) and match probability (MP) also had robust values (Figure 1B; Table S3). These findings underscore the robust performance of the YHseqY3000 panel in haplogroup classification and genetic variation capture, highlighting its effectiveness in detailed genetic profiling.

Using HaploGrouper, we classified haplogroups according to the International Society of Genetic Genealogy (ISOGG) Y-DNA Haplogroup Tree 2019–2020 (v15.73), identifying 545 definitive haplogroups among 5020 unrelated samples. We assessed the geographical distribution of major haplogroups at the third level, generating the HFS for the Han Chinese population (Figure 1C). Several founding lineages, or dominant haplogroups, were found to contribute significantly to the paternal gene pool of the Han Chinese population, with haplogroup O2a comprising 52.85%, followed by O1a at 12.47% and O1b at 9.86% (Table S4). The geographical distribution of the haplogroups revealed that O2a had the highest frequency in the northern and northeastern regions, including Heilongjiang (68.47%), Hubei (57.92%), and Anhui (56.78%) (Figure 1D). The majority of its sublineages consisted of O2a2b1a1-M117 (16.91%), O2a2b1a2a1a-F46 (9.92%), and O2a1b1a1a1a-F11 (10.66%). O1a-M119 predominated in southern and southeastern China, particularly in Zhejiang (25.98%), Jiangxi (23.47%), and Fujian (20.42%) (Figure 1E). The sublineage O1a1a1a1a1a1a1-F492 (5.40%) was notably prevalent in these areas. Conversely, O1b-M268 was predominantly found in southern China, such as Guangxi (24.72%), Guangdong (19.89%), and Hunan (14.65%) (Figure 1F). The sister subhaplogroups O1b1a1-PK4 and O1b1a2-Page59 demonstrated a latitude-dependent gradient, with O1b1a1-PK4 being more prevalent in the southern region and O1b1a2-Page59 in the northern region (Figure S1). The detailed mapping of the O haplogroup distributions across the Han Chinese population reveals regional prevalence and intricate genetic landscapes shaped by historical migration and demographic patterns.

C2b was predominantly observed in northern China, including Hebei (15.21%), Shandong (13.97%), and Beijing (13.53%) (Figure 1G). Conversely, C2a, while also common in northern regions such as Inner Mongolia (12.20%) and Tianjin (11.59%), was less common in southern China than C2b (Figure 1H). N1b was primarily found in southern and southwestern China, including Yunnan (8.06%), Chongqing (7.07%), and Shanghai (6.57%) (Figure 1I). N1a, however, was more prevalent in northern China, particularly in Shaanxi (6.32%) and Inner Mongolia (4.88%), with a notable presence in Yunnan (4.84%) (Figure 1J). D1a displayed a high frequency around the Qinghai-Tibet Plateau [41]. It was prevalent among the surrounding areas of the Qinghai-Tibet Plateau and was evident in Inner Mongolia (9.76%) and Gansu (6.15%) (Figure 1K), indicating significant interactions between Han Chinese and Tibeto-Burman-speaking populations. In northern China, Q1a was primarily found, especially in Inner Mongolia (7.32%) and Shaanxi (5.06%), with sublineages such as Q1a1a-M120 accounting for 2.75% (Figure 1L). The rare, deep-rooted haplogroup F was exclusively noted in southwestern China, aligning with the southern route of the ancient northward migration of modern humans into East Asia [36]. Additionally, non-Chinese-specific haplogroups such as R, E, G, H, I, J, and L were predominantly observed in northern and northwestern China (Table S4), suggesting Holocene trans-Eurasian connections and historical migration along the ancient Silk Road [4]. Overall, the complex paternal lineage composition of the Han Chinese population and the geographically specific distribution of certain haplogroups underscore the profound influence of historical migrations of source populations on their genetic landscape.

### Isolation-enhanced genetic differentiation influenced the paternal genetic architecture of the Han Chinese population

**Figure 2:**
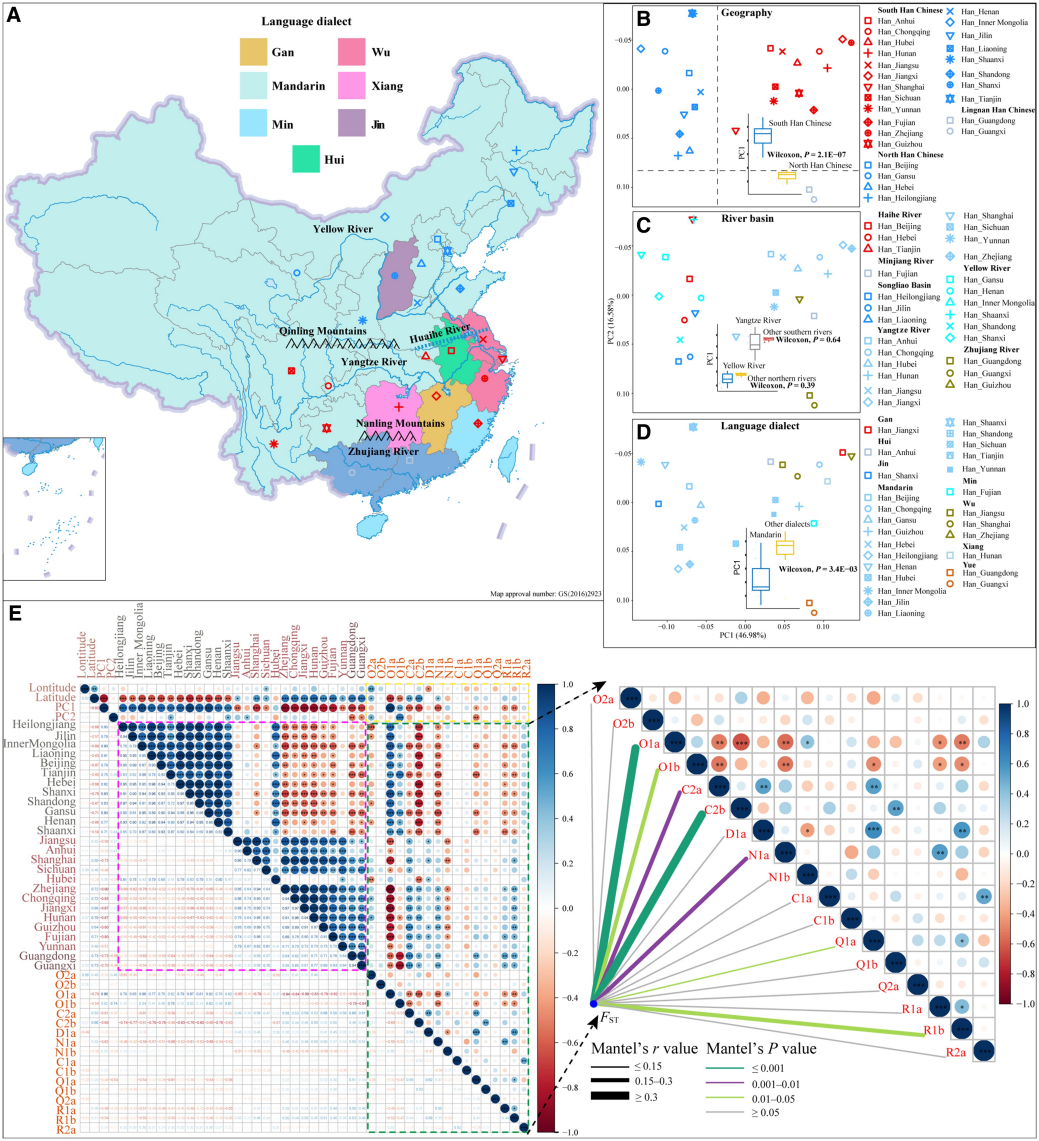
Paternal genetic structure and correlation analysis **A**. Map of the geographic barriers and river valleys. The colors of provinces indicate different language dialects. The symbols in the map correspond to those in (B). **B**. PCA of the Han Chinese populations based on the fourth-level haplogroup frequency matrix, excluding Xinjiang, Ningxia, and Hainan due to sampling bias. Populations are grouped by the geographical boundaries of the Qinling-Huaihe line and the Nanling Mountains. **C**. PCA of the Han Chinese populations based on the fourth-level haplogroup frequency matrix. Populations are grouped by river valleys. **D**. PCA of the Han Chinese populations based on the fourth-level haplogroup frequency matrix. Populations are grouped by dialect groups. **E**. Pearson’s correlation analysis between longitude, latitude, PC1, PC2, pairwise genetic distance, and haplogroup frequency among Han Chinese individuals. *, *P* < 0.05; **, *P* < 0.01; ***, *P* < 0.001. Pairwise genetic distance (*F*_ST_) was analyzed via the Mantel test (right), with edge color indicating statistical significance and edge width representing Mantel’s R statistic. PCA, principal component analysis.

To investigate genetic relatedness among the Han Chinese populations, we conducted principal component analysis (PCA) on 4987 individuals from 26 provincial-level administrative regions in China, using haplogroup frequencies at the fourth level (**Figure 2**A–D). Populations grouped by geography revealed that PC1 effectively separated Han Chinese into northern and southern clusters, with the Qinling-Huaihe line acting as a geographical boundary (Figure 2A and B). A significant correlation was detected between PC1 and latitude (*r* = **−**0.82, *P* < 0.001), whereas no significant association was detected with longitude (Figure 2E). Notably, longitude was strongly correlated with the frequency of haplogroup O2a (*r* = 0.56, *P* < 0.001), which might have resulted from the recent westward migration of Han Chinese individuals since 1949 [42] (Figure 2E, Figure S2). Furthermore, statistically significant correlations were observed between PC1 and the frequencies of O1a (*r* = 0.96, *P* < 0.001) and C2b (*r* = **−**0.69, *P* < 0.001) (Figure 2E). O1a exhibited a high frequency in southern Han Chinese populations, clustering on the right of the PCA plot, whereas its frequency decreased in northern populations on the left. Conversely, C2b showed the opposite distribution pattern. This North-South stratification was further consistently corroborated by pairwise genetic distances (*F*_ST_), a neighbor-joining tree, and correlations between genetic distances across 26 Han Chinese populations (Figure 2E, Figure S3).

The southern Han Chinese population exhibited a distinct substructure, with PC2 clearly distinguishing the Guangdong and Guangxi populations from other southern Han groups, using the Nanling Mountains as a geographic boundary (Figure 2A and B). Consequently, individuals from Guangdong and Guangxi were classified as Lingnan Han in subsequent analyses. Notably, the pairwise genetic distance between the northern and southern Han populations (*F*_ST_ = 0.01018) was comparable to that between the southern Han population and the Lingnan Han population (*F*_ST_ = 0.00973), further supporting the classification of the Lingnan Han population as a distinct genetic subclade (Table S5). The PC2 coordinates were not correlated with longitude or latitude but were strongly correlated with the O1b frequency (*r* = 0.74, *P* < 0.001) (Figure 2E). The neighbor-joining tree revealed that the Central Han population (Anhui, Jiangsu, and Shanghai) clustered together (Figure S3B), although it was not distinctly separated from other southern Han groups on the PCA plot (Figure 2B). These genetic similarity and difference patterns were confirmed through multidimensional scaling (MDS) analysis (Figure S4). Furthermore, the paternal genetic structure exhibited no isolation-by-distance pattern (*r* = 0.0006, *P* = 0.462), in contrast to the results obtained from the Mantel test for autosomal DNA [43]. The differing genetic patterns inferred from autosomal and uniparental markers highlight the varying influences of patrilocality and matrilocality residence models on the Han Chinese gene pool. Additionally, we explored the genetic affinities among Han Chinese individuals separated along different river valleys and language dialects (Figure 2C–E). Populations from the northern rivers (YRB and other northern rivers) exhibited significant differentiation from others from the southern rivers (Yangtze River and other southern rivers) along PC1 (Figure 2C). Mandarin-speaking populations were also found to differ significantly from others along PC1 (Wilcoxon rank sum test, *P* = 3.40 × 10^−^^3^) (Figure 2D), indicating an association between cultural dialects and Y-chromosome founding lineages. In conclusion, large-scale Y-chromosome sequence analysis revealed three paternal population substructures: North Han, South Han, and Lingnan Han.

To further investigate fine-scale genetic differentiation and the influence of geographical and sociocultural factors on the paternal genetic structure of Han Chinese, an analysis of molecular variance (AMOVA) was conducted, which classified geographically distinct populations into groups based on geographical region, river valley, and language dialect (**Table 1**, Table S6). Genetic variance among populations accounted for 1.02% (*P* < 0.01) (Table 1). Greater genetic homogeneity was observed in the northern Han population (0.18%, *P* < 0.01) than in the southern Han population (0.67%, *P* < 0.05), indicating distinct patterns of genetic differentiation between these geographically diverse regions (Table 1). Significant among-group variation (1.14%, *P* < 0.01) was observed when Han Chinese individuals were categorized into North Han, South Han, and Lingnan Han, which exceeded the variance observed in other groupings, such as those based on geographical regions, river valleys, or language dialects (Table 1). Notably, the among-group variation for the three river valleys presented a relatively high value (1.09%, *P* < 0.001), comparable to that of the northern and southern Han groups (1.06%, *P* < 0.001). These results showed that isolation-enhanced genetic differentiation, as indicated by geographic boundaries (the Qinling-Huaihe line and the Nanling Mountains) and river valleys (Yellow River, Yangtze River, and Zhujiang River), played a pivotal role in shaping the paternal population structure of Han Chinese individuals.

**Table 1:** Summary of the AMOVA results

Grouping	Number of populations	Number of groups	Percentage of variations (%)		
			Among groups	Among populations within groups	Within populations
Total	26	1	-	1.02**	98.98**
Southern China *vs*. Northern China^a^	26	2	1.06**	0.47**	98.46**
Southern China *vs*. Northern China *vs*. Southwestern China *vs*. Northwestern China^b^	26	4	0.88**	0.44**	98.68**
Central China *vs.* Eastern China *vs.* Northeastern China *vs.* Northern China *vs.* Northwestern China *vs*. Southern China *vs.* Southwestern China^c^	26	7	0.31	0.71**	98.98**
South Han Chinese *vs*. North Han Chinese *vs.* Lingnan Han Chinese^d^	26	3	1.14**	0.32**	98.54**
Yangtze River *vs*. Yellow River *vs.* Zhujiang River *vs.* Haihe River *vs*. Songliao Basin *vs.* Minjiang River^e^	26	6	0.96**	0.31**	98.73**
Yangtze River *vs*. Yellow River *vs.* Zhujiang River^f^	26	3	1.09**	0.32**	98.59**
Language dialect^g^	26	8	0.93**	0.36**	98.71**
Southern China^a^	14	1	-	0.67*	99.33*
Northern China^a^	12	1	-	0.18**	99.82**
Lingnan Han Chinese^d^	2	1	-	0.17	99.83
Central Han Chinese	3	1	-	−0.12	100.12

*Note*: The superscript characters indicate different groupings, as detailed in Table S6.

* *P* < 0.05;

** *P* < 0.01. AMOVA, analysis of molecular variance.

### Genetic connections across founding populations with different Han-related paternal lineages

**Figure 3:**
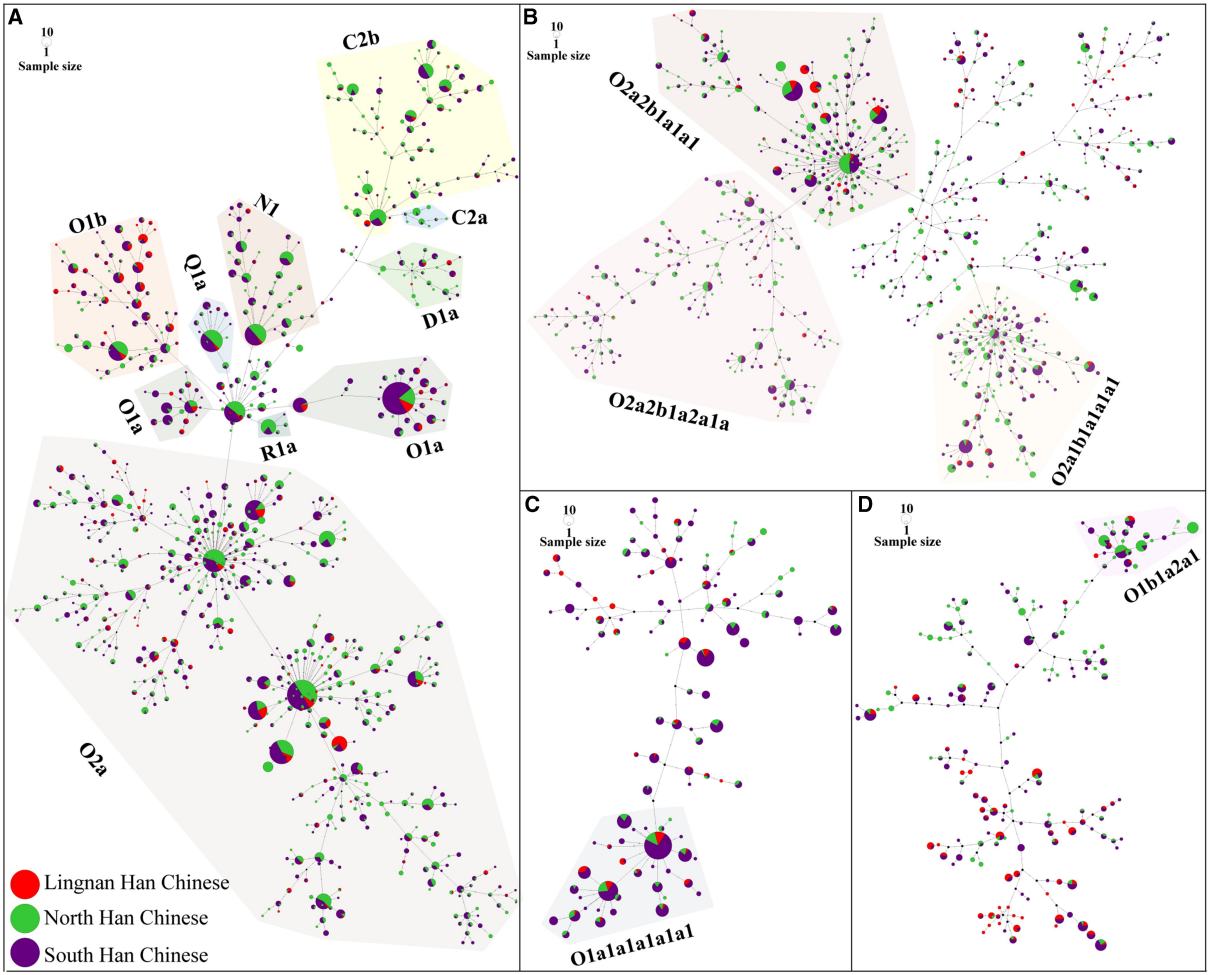
Median-joining network among Han Chinese individuals **A**. Median-joining network of all Han Chinese individuals included in the study. **B**. Median-joining network of Han Chinese individuals classified as O2a and its sublineages. **C**. Median-joining network for individuals belonging to O1a and its sublineages. **D**. Median-joining network for individuals within O1b and its sublineages. Different node colors represent different groups, whereas different background colors indicate haplogroups and subhaplogroups.

To comprehensively investigate the genetic connections among different paternal lineages and observed population substructures, a median-joining network analysis and a parsimony phylogenetic tree were constructed using 5020 Y-chromosome sequences (**Figure 3**, Figure S3). We observed that nearly every central node and branch was contributed by geographically diverse populations, and the paternal lineages consisting of O2a, O1a, O1b, and C2b contributed to most of the paternal genetic framework (Figure 3A, Figure S5). Notably, the O1a-related nodes and branches were predominantly composed of individuals from the South Han population, whereas Lingnan Han contributed primarily to the O1b-related nodes and branches. In contrast, the C2b-related nodes and branches were associated primarily with the North Han population (Figure 3A, Figure S5).

A focused network analysis was subsequently conducted on lineages O2a, O1a, O1b, C2b, C2a, N1a, N1b, Q1a, and D1a to explore the fine-scale genetic structures of these lineages in greater detail (Figure 3B and C, Figures S6–S8). The results revealed a star-like network of O2a2b1a1a1 (downstream of O-M117) and O2a1b1a1a1a1 (downstream of O-F11) (Figure 3B), suggesting the expansion of these lineages within the Han Chinese population. In contrast, no distinct hierarchical expansion structure was detected within O2a2b1a2a1a-F46, and the cluster’s origin was not central (Figure 3B), potentially indicating that the expansion of O2a2b1a2a1a-F46 occurred immediately after its emergence. O1b1a2-Page59 was observed at a high frequency in Northeast China (Figure S1), with its sublineage O1b1a2a1-F1759 displaying a star-like network in the Han Chinese population (Figure 3D). Previous population studies have indicated that O1b1a2-Page59 is primarily spread among Sinitic-speaking populations but is absent in other ethnolinguistic groups [22,23]. Additionally, O1b1a2 was identified in Neolithic individuals from the Wanggou site (5500–5000 BP) associated with Yangshao culture [11], suggesting its role in the formation of the Han Chinese population and its emergence as one of their founding paternal lineages. Similarly, the sublineages of Q1a and N1a exhibited comparable patterns (Figures S7B and S8A).

### Effects of admixture-introduced genetic differentiation on the formation of Han Chinese

The paternal substructures observed in the Han Chinese population delineated the level of genetic differentiation and extent of Y-chromosome variation, providing insights into the differences in the composition of founding lineages. Fisher’s exact test revealed that O1a, O1b, C2b, C2a, R1a, R1b, N1a, and J2a presented significant differences in haplogroup frequency distribution between southern and northern Han populations (Table S7). A Mantel test was subsequently conducted to assess the correlation between pairwise genetic distances and haplogroup composition (Figure 2E). The results indicated that O1a, O1b, C2b, C2a, N1a, and R1b were major contributors to the genetic distance discrepancies between Han Chinese subpopulations (Figure 2E). Spatial autocorrelation analysis revealed regional clustering patterns among these primary haplogroups, potentially reflecting their diffusion centers (Figure 1D–I). Specifically, O1a and O1b exhibited strong clustering in southern China, with O1a concentrated near the Yangtze River and O1b in the Lingnan region (Figure 1E and F). In contrast, significant clustering of C2b and C2a was observed in northern China (Figure 1G and H). However, no clear regional clustering was detected for the widespread haplogroup O2a and its primary subhaplogroups O-M117, O-F46, and O-F11, suggesting extensive admixture among the three Neolithic Super-Grandfathers (Figure 1E, Figure S1). Overall, extensive migration and admixture of ancestral populations associated with haplogroups O1a, O1b, and C2b have been identified as the primary factors influencing the paternal genetic structure of the Han Chinese population. Additionally, C2a-, N1a-, R1a-, R1b-, and J2a-related ancestral populations also contributed to the observed genetic differentiation within these populations.

**Figure 4:**
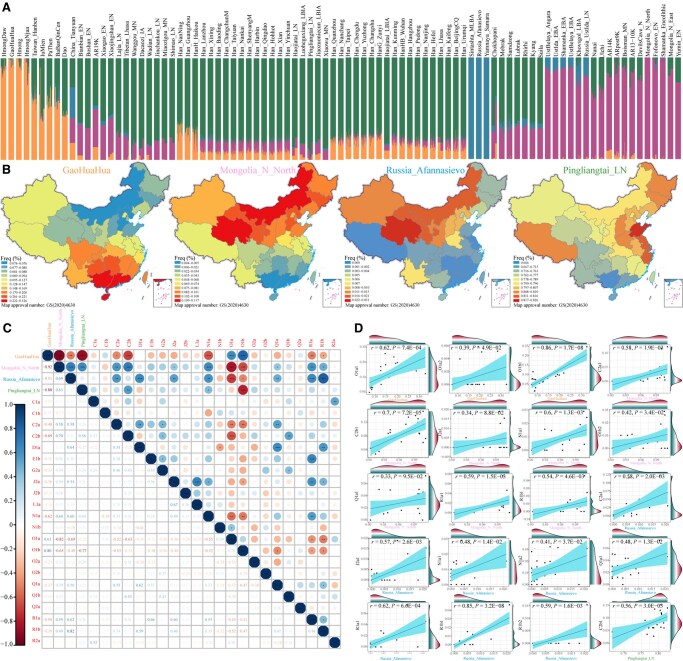
Correlation analysis between admixture ancestry components and paternal lineages among Han Chinese individuals **A**. ADMIXTURE results illustrating ancestry components from West Eurasia, Siberia, and East Asia among Han Chinese individuals. **B**. Distribution of admixture ancestry components among Han Chinese individuals. **C**. Correlation analysis between admixture ancestry components and paternal lineages at the third level. *, *P* < 0.05; **, *P* < 0.01; ***, *P* < 0.001. **D**. Pearson’s correlation analysis between admixture ancestry components and paternal lineages at the fourth level.

The paternal lineages observed in the Han Chinese population were highly diverse, with both ancient indigenous and incoming lineages contributing to their genetic differentiation. To quantify the extent of ancient Eurasian populations’ contributions to Han Chinese paternal genetic composition since the Holocene, a model-based ADMIXTURE analysis was conducted on 32 geographically diverse Han Chinese populations and ancient Eurasian populations. This analysis aimed to reveal the basic genetic admixture profiles (**Figure 4**A). The findings indicated that a four-source admixture model best explained the genetic ancestry profile of the Han Chinese population, comprising YRB-related ANEA ancestry (Pingliangtai_LN; green), ASEA-related ancestry (GaoHuaHua; orange), AEES-related ancestry (Mongolia_N_North; purple), and AWES-related ancestry (Russia_Afanasievo; blue) (Figure 4A). Notably, the distribution of these ancestral components varied significantly across geographically diverse Han Chinese populations (Figure 4B). Specifically, the proportions of individuals with AEES, AWES, and ANEA-related ancestry tended to decrease from north to south, whereas ASEA-related ancestry exhibited an inverse pattern among Han Chinese individuals (Figure 4B).

Previous genomic studies have demonstrated male-dominant, sex-biased admixture in Han Chinese, Tibeto-Burman-speaking, and Hui populations [21,44]. Consequently, if the proportion of the estimated ancestral component demonstrates a significant positive correlation with the frequency of paternal lineages, it is plausible that the expansion and admixture events related to the ADMIXTURE-inferred ancestral sources may have contributed to the incorporation of the paternal lineage into the formation of the targeted Han Chinese population. To investigate this further, we conducted a correlation analysis between the proportion of ADMIXTURE-inferred ancestry and the frequency of paternal lineages across geographically distinct Han Chinese populations. Significant correlations were identified: ASEA-related ancestry was associated with O1a and O1b; AEES-related ancestry was associated with C2a, C2b, J2a, N1a, Q1a, R1a, and R1b; AWES-related ancestry was associated with C2a, D1a, J2a, N1a, Q1a, R1a, and R1b; and ANEA-related ancestry was associated with C2b and N1a (Figure 4C). Further analysis of the fourth-level sublineages revealed that the downstream branches of these lineages also exhibited correlations with the corresponding genetic ancestry (Figure 4D). Notably, the paternal lineages introduced by these ancestral populations significantly contributed to the genetic differentiation observed in Han Chinese populations (Figure 2E; Table S7). Overall, these external paternal lineages, introduced via admixture, played a critical role in the genetic divergence between northern and southern Han Chinese, underscoring the importance of population admixture across Eurasia in shaping the paternal genetic landscape of the Han Chinese population.

### WDMSA model for the formation of the Han Chinese population

**Figure 5:**
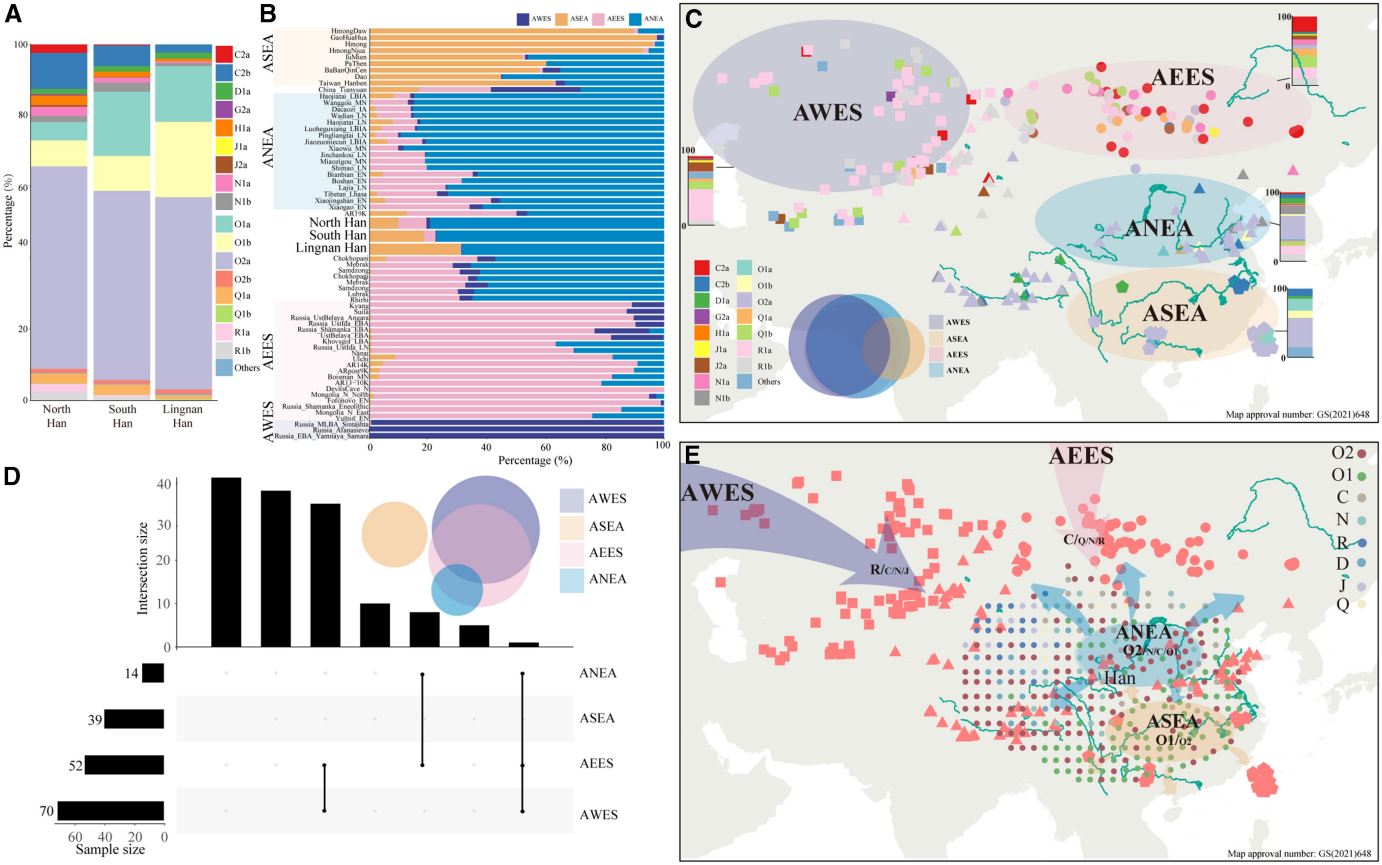
Simplified evolutionary pattern of the Han Chinese population **A**. Stacked bar plot showing the distribution of paternal haplogroups across the Han Chinese genetic substructures. Each color represents a distinct haplogroup. Haplogroups with fewer than 10 individuals are grouped as “Others” (including E1b, L1a, C1b, Q2a, R2a, I2a, J2b, T1a, Q2b, I1a, and I1c). **B**. Model-based ADMIXTURE analysis depicting ancestral component proportions among the meta-population of ancestral sources and target populations, categorized according to Han Chinese paternal substructures. **C**. Stacked bar plots illustrating the frequency of ancient Y-chromosome haplogroups associated with four defined ancestries: AEES, AWES, ANEA, and ASEA. Different shapes denoted different categories. A corresponding Venn diagram visualizes haplogroup-sharing patterns in ancient DNA. **D**. UpSet plot highlighting haplogroup sharing across the four ancestry categories. Only haplogroups showing statistically significant positive associations (*P* < 0.05) with respective ancestry components are shown, spanning haplogroup levels 3 to 10. **E**. Schematic model proposing a weakly differentiated, multi-source admixture process underlying Han Chinese population formation. Circles denote haplogroups; shapes in red are consistent with categories. Ancient DNA was also labeled here. ANEA broadly refers to northern East Asian groups with YRB ancestry. Pingliangtai_LN and related Yangshao-derived populations serve as proxies, reflecting millet-farming lineages and ancestral components associated with both the Yellow River region and ancient populations from Xizang. ASEA represents southern East Asian rice-farming populations. GaoHuaHua serves as the proxy. AEES refers to ancestry from eastern Eurasia, particularly steppe hunter-gatherers. Mongolia_N_North is designated as the proxy, capturing ancient Northeast Asian influence. AWES links to western Eurasian steppe herders, especially those of the Afanasievo culture, which serves as the proxy. AEES, Ancient Eastern Eurasian Steppe; AWES, Ancient Western Eurasian Steppe; ANEA, Ancient Northern East Asian; ASEA, Ancient Southern East Asian; YRB, Yellow River Basin.

Genetic analyses demonstrated that multiple sources contributed to the genetic diversity of the Han Chinese population, as indicated by both autosomal ancestral components and paternal lineages (**Figure 5**A and B). An examination of ancient paternal lineages revealed shared paternal lineages among the four identified ancestral populations since the Holocene (Figure 5C). For example, haplogroups R1a and R1b, typically associated with AWES-related populations, were also found in AEES-related populations, such as Mongolia_LBA_MongunTaiga_3 (R1a) and Mongolia_EBA_Chemurchek (R1b) [10,45]. Similarly, N1a appeared in both AWES-related (Mereke_MBA) and AEES-related (Munkhkhairkhan_MBA) populations. The ANEA-related lineage O2a was detected in individuals (Mongolia_LBA_CenterWest_5) from AEES-related populations [10,11], whereas C2b was detected in both AEES-related (Russia_Siberia_Lena_EN) and ANEA-related (Shimao_LN and Miaozigou_MN) populations [11,46]. Furthermore, the correlation between lineage frequency and ADMIXTURE-based ancestry proportions indicated that multiple ancestral sources contributed identical paternal lineages to the Han Chinese gene pool (Figure 5D). R1a, R1b, and N1a were associated with AWES/AEES-related ancestry components, whereas C2b was linked to the AEES/ANEA-related ancestry components (Figure 4C). These findings align with current paleogenomic data from spatiotemporally diverse Eurasian populations. The shared and correlated paternal lineages suggest potential gene flow between these ancestral populations.

The admixture patterns of the Han Chinese population were further examined through autosomal genomic variation to investigate the ancient divergence and recent connections of ancestral sources in its formation. ADMIXTOOLS2 analysis was employed to assess population divergence and gene flow between Han Chinese and ancient populations, leading to the identification of an admixture model that best explained Han Chinese formation (Figure S9). The optimal admixture graph indicated that 51% of Han Chinese ancestry was related to GaoHuaHua and 49% to YRB millet farmers. Additionally, ancient gene flow was detected between Afanasievo herders and Mongolia Plateau hunter-gatherers, as well as between Mongolia_N_North and China_YR_LN, highlighting early connections among these putative ancestral sources. These findings suggest that ancient East Asians, including ANEA and ASEA populations, as well as ancient Siberians and western Eurasian pastoralists with differentiated yet connected genetic lineages, contributed to the formation of the modern Han Chinese population (Figure 5E). The native ancestry sources, represented by ANEA- and ASEA-related populations, were primarily the O2 and O1 haplogroups and their sublineages, which had historical connections to the founding lineages of cultivated millet and rice farmers. Siberian hunter-gatherers with AEES-related ancestry components disseminated C and its sublineages primarily through complex interactions and admixture with AWES-related populations and ANEA-related millet farmers. AWES-related pastoralists mainly brought R and its subhaplogroups into northwestern Han Chinese with pastoralist expansion or Holocene trans-Eurasian cultural exchange and population admixture, which also exhibited a connection with AEES-related populations. Hence, we propose the WDMSA model to explain the formation of Han Chinese, which summarizes the weakly differentiated but interconnected ancestral sources resulting from extensive expansion and admixture processes (Figure 5). The model suggests that multiple, complex, and multilayered ancestral sources contributed to the formation of geographically diverse modern Han Chinese populations. Our identified fine-scale paternal genetic structure also suggests that both isolation-enhanced and admixture-introduced genetic differentiation contributed to the genetic differences among the North, South, and Lingnan Han Chinese populations.

### Genetic relatedness between the Han Chinese population and other ethnolinguistic populations in East Asia

**Figure 6:**
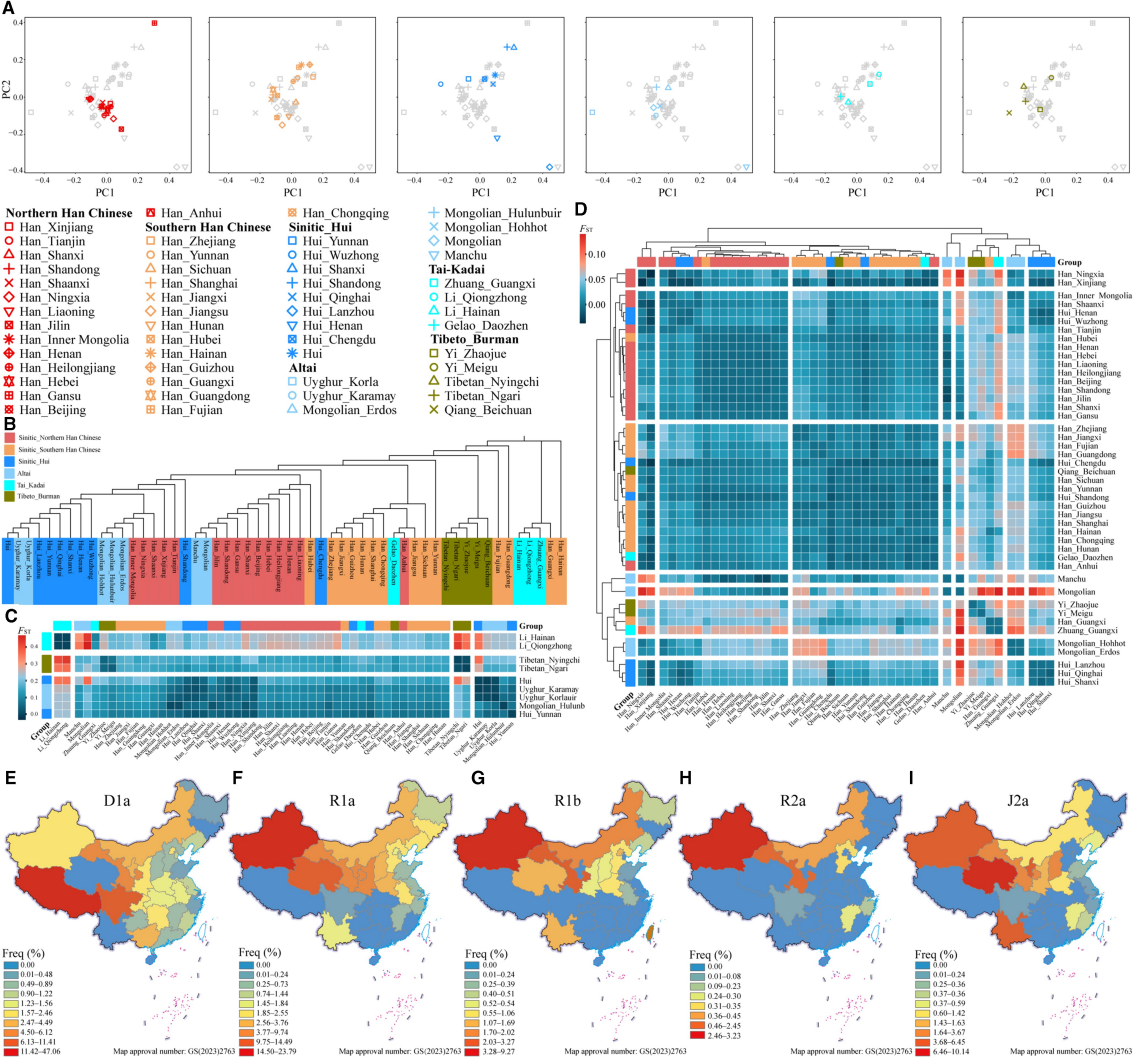
Genetic relationships among East Asians inferred from Y-chromosome variations **A**. PCA inferred from the haplogroup frequency matrix of 54 ethnolinguistically diverse populations in East Asia. **B**. Neighbor-joining tree of 54 ethnolinguistically diverse populations in East Asia. **C**. Heatmap of pairwise genetic distances (*F*_ST_) among 54 ethnolinguistically diverse populations (broad-scale visualization). **D**. Heatmap of pairwise genetic distances (*F*_ST_) among 54 ethnolinguistically diverse populations (fine-scale visualization). **E**.–**I**. Haplogroup frequencies of D1a (E), R1a (F), R1b (G), R2a (H), and J2a (I) among 54 ethnolinguistically diverse populations in East Asia.

To investigate the genetic affinity and admixture signatures between Han Chinese and other ethnolinguistic populations in East Asia, we assembled a comprehensive dataset of 10,481 individuals from 54 populations representing four language families by aggregating our data with published haplogroup information [22,23,39]. PCA based on fourth-level haplogroup frequency was performed to elucidate the genetic relationships among these populations. A distinct separation between the northern and southern Han Chinese populations was observed along the PC2 axis (**Figure 6**A), which was further supported by the neighbor-joining tree and the distance matrices (Figure 6B–D). Interestingly, the clustering of populations appeared to correlate with linguistic affiliation (Figure 6B–D), suggesting potential co-diffusion between language families and paternal lineages. The Altaic-speaking populations of northern China were found to be genetically close to northern Han Chinese, whereas the Tibeto-Burman/Tai-Kadai-speaking populations from southern China were relatively close to southern Han Chinese (Figure 6B–D). These patterns indicate potential gene flow between Han Chinese individuals and surrounding ethnolinguistically diverse populations. Notably, the Hui population clustered closely with the Han Chinese population, which is consistent with previous findings [44]. A high frequency of haplogroup D1a was observed in Tibetans. Haplogroups R1a, R1b, and R2a were predominantly distributed in northwestern China, especially Xinjiang, reflecting historical gene flow between West and East Eurasians. Haplogroup J2a was found at high frequency in Qinghai, which is located within the Hexi Corridor, potentially indicating the influence of the ancient Silk Road in connecting China with the West [47,48]. These findings provide new insights into the complex interactions and gene flow dynamics between Han Chinese and ethnolinguistically diverse populations in East Asia, as well as between ancient West and East Eurasians.

## Discussion

Large-scale genetic studies of autosomal DNA have significantly advanced the understanding of the fine-scale genetic structure and the demographic history of East Asians [49,50]. Y-chromosome and mitochondrial DNA (mtDNA) variations have provided complementary insights into their sex-specific genetic history [21,34,35,44]. However, previous Y-chromosome studies in East Asia have focused predominantly on a limited set of Y-SNPs and regional Han populations, thereby restricting the exploration of the broader paternal genetic structure of Han Chinese individuals and hindering a comprehensive understanding of the role of Y-chromosome variations in shaping their genetic framework. To address this gap and enhance the understanding of the fine-scale demographic history of Han Chinese individuals, we established the YanHuang cohort. This pilot work presents Y-chromosome variation profiles from 5020 Han Chinese individuals across 29 provincial-level administrative regions via the YHseqY3000 panel. The high resolution of haplotype- and haplogroup-based parameters demonstrates the robust performance of the YHseqY3000 panel in haplogroup allocation and variation capture. Furthermore, one of the most comprehensive HFS for the Han Chinese population is presented, with O2a, O1b, O1a, and C2b accounting for the majority of the haplogroup composition. Notably, significant paternal genetic differentiation was observed at the geographic boundaries of the Qinling-Huaihe line and the Nanling Mountains, alongside evidence of population admixture between western and eastern Eurasian populations and northern and southern East Asian populations.

### Isolation-enhanced genetic differentiation in paternal genetic structure

This study revealed clear North-South genetic divergence within the Han Chinese population, which is consistent with prior findings based on autosomal DNA and mtDNA [21,30]. Furthermore, three distinct subgroups were observed, including North Han, South Han, and Lingnan Han, corresponding to geographical divisions (Figure 2B). Previous research on autosomal variations identified four subgroups: North Han, South Han, Central Han, and Lingnan Han, with the Central Han population exhibiting closer genetic proximity to the North Han population than to the South Han and Lingnan Han populations [31]. However, our analysis revealed that the Central Han population was more closely related to the South Han population (0.0022, *P* < 0.01) than to the North Han population (0.0089, *P* < 0.01) from a paternal perspective (Table S5). This divergence in genetic structure suggests that Y-chromosome and autosomal variations reflect different aspects of genetic history.

The AMOVA-based results indicated that the greatest among-group variation (1.14%, *P* < 0.01) was observed when populations were grouped by geographical boundaries (Table 1), further supporting the presence of genetic stratification. Northern Han Chinese individuals presented greater genetic homogeneity, whereas southern Han Chinese individuals presented greater diversity (Table 1). This increased diversity may be attributed to prehistoric migrations and interactions between southern Han Chinese and indigenous populations [21,36]. The Qinling-Huaihe line, which marks a division in hydrology and climatology, roughly corresponds to the transition between millet-based agriculture in the YRB and rice-based farming in the Yangtze River Basin [51]. The Nanling Mountains act as geographical barriers between the Yangtze River and Zhujiang River Basins. This may have led to the relatively elevated degree of differentiation observed among the Yangtze River, Yellow River, and Zhujiang River populations.

Previous studies have established a correlation between language dialects and the autosomal DNA-inferred genetic landscape [17,33], although such correlations are less common for mtDNA [38]. In our study, genetic differences were observed between Han Chinese individuals who speak Mandarin and those who speak other dialects (Figure 2D), a pattern that may be attributed to patrilocal residence customs in East Asia [52]. Notably, genetic divergence was detected only between Mandarin speakers and other dialect groups. The observed paternal genetic structure and divergence suggest that geographic isolation has played a crucial role in shaping the paternal genetic landscape of the Han Chinese population. Additionally, river basins are linked to agricultural traditions, which are strongly related to population expansion and have been proven to influence the maternal genetic landscape of the Han Chinese. Future whole-genome sequences of the Y chromosome may provide further insight into whether agricultural practices have also impacted the paternal genetic landscape.

### Expansion events related to the paternal founding lineages among Han Chinese

We identified multiple expansion events related to paternal founding lineages, such as O-M117, O-F11, and O-F46 (Figure 3). The phylogenetic bifurcations of three Neolithic Super-Grandfathers of Han Chinese were reconciled, revealing deeper branch expansions [53]. Ancient DNA analysis revealed O1a in the 5300- to 4100-year-old Liangzhu population from the Yangtze River delta [54]. Previous studies have demonstrated that O1a sublineages have experienced a complex demographic history over the past 10,000 years, with Neolithic communities in Southeast China contributing to the gene pool of Sinitic, Tai-Kadai, and Austronesian-speaking populations. The pronounced star-like network of O1a1a1a1a1a1-F492 suggests a significant expansion event of this haplogroup in the Han Chinese population, which is consistent with the phylogenetic radiation reported in earlier studies [55]. These findings highlight internal genetic connections and expansion events within the Han Chinese population. Moreover, the consistency of these events with prior phylogenetic research demonstrates that our designed panel can comprehensively capture the genetic diversity of Han Chinese populations.

### Effects of admixture-induced genetic differentiation on paternal genetic structure

Admixture is a key driver of genomic diversity, reducing between-population differentiation while increasing within-population genetic variation. In this study, Fisher’s exact test and the Mantel test identified haplogroups O1a, O1b, C2b, C2a, N1a, R1a, R1b, and J2a as contributing to paternal genetic differentiation (Figure 2E; Table S7). These haplogroups were also correlated with the four ancestral components simulated by ADMIXTURE (Figure 4C), suggesting that admixture played a central role in their introduction. Although some correlations, such as those involving D1a and AWES-related populations, may be affected by sample bias and the scarcity of ancient DNA in East Asia, most significant correlations are supported by archaeogenetic evidence. The appearance of these haplogroups in corresponding ancient populations further supports their relevance, although the precise timing of admixture events remains challenging to establish. Notably, the O2a haplogroup, which was frequently observed in ANEA-related populations, dominated the paternal genetic landscape but did not contribute to genetic divergence. This pattern may indicate that the demographic expansion of the Han Chinese population from north to south was driven primarily by ancient populations bearing the O2a haplogroup.

The Han Chinese population can be viewed as a tapestry shaped by numerous admixture events. Historical records suggest that Han Chinese are primarily descendants of the ancient Huaxia tribe, which was originally located in the upper and middle reaches of the YRB. Approximately 6000 years ago, the proto-Han Chinese diverged from the proto-ST people, migrating into southern and eastern East Asia. Prolonged migration, interaction, and admixture with surrounding populations facilitated the growth of Han Chinese into the largest population with the broadest distribution in China. In our study, multiple ancestral sources from distinct Eurasian regions were identified, contributing to the complex paternal gene pool of Han Chinese. This finding aligns with our proposed WDMSA model, which involves weakly differentiated and interconnected ancestral sources. Ancient DNA studies support this complexity. For example, Yang et al. reported gene flow between northern and southern China since the Neolithic, with greater genetic affinity observed in the present than in the past [12]. Wang et al. identified contributions of northern ancestry to ancient farmers in Taiwan, China during the Neolithic [10]. Similarly, Jeong et al. documented gene flow from western pastoralists into eastern Eurasia at approximately 3000 BCE [14]. While the WDMSA model offers general insights into Han Chinese formation, the history of migration and admixture is multiwave and multilevel. Future studies with broader sampling, higher-resolution sequences, and more paleogenomic data are expected to reveal a more detailed evolutionary history of the Han Chinese population.

### Limitations

The measurement of Y-chromosome complex regions, particularly telomeric, centromeric, and segmental duplication regions, has presented challenges in recent years [56]. This study focused on high-confidence phylogenetically informative markers in the male-specific areas of the Y chromosome, utilizing capture target sequencing to reveal the paternal structure of Han Chinese populations comprehensively. Advances in third-generation long-read sequencing technologies, such as Oxford Nanopore and PacBio HiFi, along with innovations in statistical assembly and genotyping, have provided greater clarity regarding the complete sequence and critical features of the Y chromosome, including its pseudoautosomal regions, X-degenerate regions, ampliconic palindromic regions, q-arm heterochromatin, and centromeric satellites [57,58]. Future studies should incorporate high-coverage, whole Y-chromosome sequences from genetically diverse cohorts, including the YanHuang project, 10K_CPGDP, and other regionally representative populations, to deepen our understanding of the Y-chromosome landscape in Han Chinese [3,6]. Additionally, sampling bias, particularly from high-altitude regions such as the Qinghai-Tibet Plateau, remains a concern. The WDMSA model introduced here offers refined insights into the paternal history of Han Chinese populations, improving upon earlier demic diffusion models derived from limited Y-chromosome and mtDNA variations [21]. Nonetheless, testing a broader range of evolutionary scenarios will require uniformly high-coverage data from all representative Chinese populations. Finally, anthropologically informed sampling strategies that encompass both Han Chinese and minority ethnic groups are essential for capturing the full spectrum of ethnolinguistic diversity and mitigating interpretive biases related to underrepresented populations.

## Conclusion

We reported the largest paternal genomic dataset from the YanHuang cohort, comprising extensive data from 5020 Han Chinese individuals across 29 geographically distinct populations. We identified fine-scale paternal genetic structures, including those of the North, South, and Lingnan Han populations, with clear differentiation along the Qinling-Huaihe line and the Nanling Mountains. The frequency distributions of multiple complex founding lineages revealed significant latitude-dependent changes across these populations. Notably, we identified two major evolutionary forces — genetic differentiation driven by isolation and admixture — that play pivotal roles in shaping paternal genetic structure. Additionally, we introduced the WDMSA model to explain the complex, multilayered ancestral contributions from local millet and rice farmers, as well as neighboring Siberian hunter-gatherers and herders, to the genomic landscape of different Han Chinese populations. In conclusion, we offered new insights into the paternal genetic structure of Han Chinese individuals, highlighting the critical factors involved in their formation, including isolation-enhanced and admixture-introduced genetic differentiation, and we proposed a comprehensive admixture model to elucidate their evolutionary history.

## Materials and methods

### Study populations

Peripheral blood samples were collected from 5020 unrelated males across 29 provincial-level administrative regions (Figure 1A), including Anhui (*n* = 236), Beijing (*n* = 133), Chongqing (*n* = 99), Fujian (*n* = 142), Gansu (*n* = 65), Guangdong (*n* = 387), Guangxi (*n* = 89), Guizhou (*n* = 64), Hainan (*n* = 11), Hebei (*n* = 230), Heilongjiang (*n* = 92), Henan (*n* = 253), Hubei (*n* = 183), Hunan (*n* = 198), Inner Mongolia (*n* = 41), Jiangsu (*n* = 499), Jiangxi (*n* = 196), Jilin (*n* = 69), Liaoning (*n* = 161), Ningxia (*n* = 12), Shaanxi (*n* = 158), Shandong (*n* = 594), Shanghai (*n* = 137), Shanxi (*n* = 158), Sichuan (*n* = 342), Tianjin (*n* = 69), Xinjiang (*n* = 10), Yunnan (*n* = 62), and Zhejiang (*n* = 331). Genomic DNA was extracted via the QIAamp DNA Micro Kit (Catalog No. 56304, QIAGEN, Hilden, Germany). DNA concentrations were quantified with the Qubit dsDNA HS Assay Kit (Catalog No. Q32854, Thermo Fisher Scientific, Waltham, USA) following the standard protocol on a Qubit 3.0 fluorometer (Thermo Fisher Scientific). The extracted DNA samples were stored at –20°C until amplification.

### Library preparation, sequencing, mapping, and genotyping

Libraries were prepared via the MultipSeq Custom Panel (iGeneTech, Beijing, China) following the manufacturer’s protocol. A DNA input of 5 ng was recommended. DNA amplification was performed via the ProFlex-96 Well PCR System (Thermo Fisher Scientific), followed by purification of the PCR products with Agencourt AMPure XP beads (Catalog No. A63881, Beckman Coulter, IN). Product quantification was carried out via the Invitrogen Qubit 3.0 Fluorometer, while purity and fragment length were assessed via the Qsp400 Bio-Fragment Analyzer (BiOptic, Taiwan, China). Libraries were paired-end sequenced (2 × 150 bp) on the Salus Pro gene sequencer (Shenzhen Salus BioMed, China). The raw FASTQ data were processed with Trimmomatic (v0.38) [59] to remove adapters and trim sequences. Sequence alignment to the GRCh37 human reference genome was conducted via BWA-MEM (v0.7.12). Reads were sorted with SAMtools (v1.9) [60]. Variant calling was performed in haploid mode via GATK HaplotypeCaller (v3.8) [61], and GVCF files were combined with GATK CombineVariants (v3.8). Genotyping of 2999 targeted SNPs was conducted via GATK GenotypeGVCFs (v3.8). The average sequencing depth was 710.9×, with a maximum of 2444.6× and a minimum of 51.7× (Figure S10).

### Haplogroup allocation

The haplogroups were classified via the developed forensic phylogenetic tree and an in-house pipeline. In alignment with prior research on haplogroup nomenclature, each sample’s haplogroup was assigned via the Python package hGrpr2 in HaploGrouper, which is based on the ISOGG Y-DNA Haplogroup Tree (v15.73; 2019–2020) [62]. Additionally, Y-LineageTracker was employed to classify the haplogroups and assess the consistency of allocation [63]. The detailed results are provided in Table S2.

### Estimation of forensic parameters

Arlequin (v3.5.1.3) was used to calculate the haplotype frequency across 29 populations. Haplotype- and haplogroup-based forensic parameters were derived via the following formulas. Haplotype diversity (HD) was computed following the formula of Nei and Tajima: HD = $n\;(1-\Sigma {pi}^{2})/(n-1)$, where n represents the total number of observed haplotypes and pi denotes the frequency of the *i*-th haplotype. Discrimination capacity (DC) was determined as the ratio between the number of observed haplotypes and the total number of haplotypes. Match probability (MP) was calculated as the sum of the squared haplotype frequencies: MP = $\Sigma {pi}^{2}$.

### Y-chromosome-based statistical analyses

#### Genetic diversity

The Python package ClusterHaplogroup in Y-LineageTracker was employed to calculate haplogroup frequencies across 29 populations from different provinces/autonomous regions/municipalities. To mitigate potential bias due to small sample sizes (< 30), samples from Xinjiang, Ningxia, and Hainan were excluded from further analysis. HFS at the third level was generated via the R package. The geographic distribution of haplogroup frequencies was mapped via ArcGIS software, followed by spatial autocorrelation analysis. Moran’s I was applied to assess whether the spatial distribution of haplogroups exhibited clustering properties, whereas Getis-Ord Gi* was used to identify HotSpot and ColdSpot regions, indicating areas of high and low haplogroup concentrations, respectively. The HotSpot regions typically correspond to centers of haplogroup diffusion. We also analyzed a cohort of 214,307 Han Chinese males from the Huaxi Biobank, excluding province-based populations with fewer than 200 individuals, to assess the geographic distribution of major paternal haplogroups (Figure S11). The distribution of these haplogroups aligned closely with the results reported in this study.

#### Genetic relationships and population structure

PCA based on haplogroup frequency at the fourth level was performed via the Python package ClusterHaplogroup in Y-LineageTracker. Pairwise genetic distances (*F*_ST_) among the 26 populations were calculated with Arlequin (v3.5.1.3), followed by nonparametric MDS analysis via the R package [64]. Correlation analyses and Mantel tests were conducted via the R package. An unrooted neighbor-joining tree based on genetic distances was constructed among the 26 populations via MEGA (v7) [65]. A genetic distance matrix was also generated via the R package. AMOVA was carried out with Arlequin (v3.5.1.3). The Mantel test was used to examine the isolation-by-distance model, reflecting the relationship between genetic and geographic distances via the R package with 10,000 permutations.

#### Phylogeny analysis and network analysis

A Newick file representing the phylogenetic tree of all samples was generated via the Python package PhyloHaplogroup in Y-LineageTracker, employing the maximum parsimony method. The resulting Newick file was then imported into the Interactive Tree of Life (iTOL) platform for annotation and visualization. Fasta files were converted to Nexus format via the Python package fasta_to_nexus_Main.py (fasta_nexus_converter/Main.py at master · rubenAlbuquerque/fasta_nexus_convert- er · GitHub). The median-joining network was subsequently generated via popART [66,67].

### Autosome-based analyses

#### Model-based ADMIXTURE analysis

A total of 718 Han Chinese individuals from 32 provincial-level administrative regions, along with 405 ancient and modern reference individuals from 57 populations, were sampled from the 10K_CPGDP and Human Origins databases [24–28,68]. The dataset was pruned via PLINK (v1.90) to remove SNPs in strong linkage disequilibrium (LD) based on the parameters “--indep-pairwise 200 25 0.4” [69]. Unsupervised ADMIXTURE (v1.3.0), which uses a maximum likelihood clustering algorithm [70], was then applied to investigate the genetic structure and identify ancestral sources at the autosomal chromosome level across different regions in China. ADMIXTURE was run for 100 iterations with ancestral sources ranging from *K* = 2 to *K* = 20, using default parameters. The optimal *K* value was determined through 10-fold cross-validation (--cv = 10) on the basis of the lowest cross-validation error and highest log-likelihood [71]. ArcMap (v10.8) was employed to visualize the frequency distributions of the four ancestral components across 32 Han Chinese populations from various administrative provinces. These four ancestral components were further correlated with paternal lineage frequencies, with the results visualized via the R package.

#### ADMIXTOOLS2 analysis

For the ADMIXTOOLS2 analysis [72], we integrated data from 32 Han Chinese populations along with continentally representative modern and ancient source populations, including Yoruba, Russia_Afanasievo, China_SEastAsia_Island_EN, Mongolia_N_North, China_YR_LN, and GaoHuaHua. The function *find_graphs* in the R package ADMIXTOOLS2 was used to establish 0 to 2 admixture events with 50 replicates for each Han Chinese population. This approach leverages *f-*statistics, and the optimal model fit is shown in Figure S9.

## Ethical statement

The Medical Ethics Committee of West China Hospital of Sichuan University approved this study (Approval No. 2023-1321), which was conducted in accordance with the principles of the Helsinki Declaration [73]. Written informed consent was obtained from the participants.

## Supplementary Material

qzaf049_Supplementary_Data

## Data Availability

The raw sequence data reported in this study have been deposited in the Genome Variation Map [74] at the National Genomics Data Center (CNCB), China National Center for Bioinformation (CNCB) (GVM: GVM000865), and are publicly accessible at https://ngdc.cncb.ac.cn/gvm. The release of variation from 5020 samples in this work is permitted by the Ministry of Science and Technology of the People’s Republic of China (Permission No. 2025BAT00383).

## CRediT author statement

**Zhiyong Wang:** Investigation, Methodology, Data curation, Formal analysis, Software, Writing – original draft, Writing – review & editing. **Kaijun Liu:** Investigation, Formal analysis. **Haibing Yuan:** Formal analysis, Data curation. **Shuhan Duan:** Data curation, Formal analysis. **Yunhui Liu:** Data curation, Software. **Lintao Luo:** Data curation, Software. **Xiucheng Jiang:** Data curation, Formal analysis. **Shijia Chen:** Formal analysis, Visualization. **Lanhai Wei:** Methodology, Data curation. **Renkuan Tang:** Data curation, Formal analysis. **Liping Hu:** Formal analysis, Data curation. **Jing Chen:** Methodology, Formal analysis. **Xiangping Li:** Formal analysis, Visualization. **Qingxin Yang:** Formal analysis, Data curation. **Yuntao Sun:** Formal analysis, Visualization. **Qiuxia Sun:** Formal analysis, Visualization. **Yuguo Huang:** Formal analysis, Visualization. **Haoran Su:** Formal analysis, Visualization. **Jie Zhong:** Data curation, Validation. **Hongbing Yao:** Data curation, Validation. **Libing Yun:** Data curation, Validation. **Jianbo Li:** Data curation, Formal analysis. **Junbao Yang:** Formal analysis, Data curation. **Yan Cai:** Formal analysis, Data curation. **Hong Deng:** Data curation, Software. **Jiangwei Yan:** Data curation, Software. **Bofeng Zhu:** Data curation, Software. **Kun Zhou:** Formal analysis, Data curation. **Shengjie Nie:** Conceptualization, Investigation, Methodology, Project administration, Resources, Supervision, Writing – review & editing. **Chao Liu:** Conceptualization, Funding acquisition, Investigation, Methodology, Project administration, Resources, Supervision, Writing – review & editing. **Mengge Wang:** Investigation, Methodology, Data curation, Formal analysis, Software, Funding acquisition, Writing – original draft, Writing review & editing. **Guanglin He:** Conceptualization, Funding acquisition, Investigation, Project administration, Resources, Supervision, Writing – review & editing. All authors have read and approved the final manuscript.

## ORCID

0009-0001-9573-7106 (Zhiyong Wang)

0009-0006-6600-871X (Kaijun Liu)

0000-0002-3155-6760 (Haibing Yuan)

0009-0005-7214-581X (Shuhan Duan)

0009-0005-7252-5169 (Yunhui Liu)

0009-0001-5790-4866 (Lintao Luo)

0000-0001-7388-6648 (Xiucheng Jiang)

0009-0006-2269-9432 (Shijia Chen)

0000-0003-3119-7400 (Lanhai Wei)

0000-0003-0008-1288 (Renkuan Tang)

0009-0009-6938-9433 (Liping Hu)

0009-0006-1785-1188 (Jing Chen)

0009-0000-9308-0780 (Xiangping Li)

0009-0007-5099-6925 (Qingxin Yang)

0009-0007-8419-1420 (Yuntao Sun)

0009-0007-4335-6364 (Qiuxia Sun)

0000-0003-4523-2289 (Yuguo Huang)

0009-0009-9312-3432 (Haoran Su)

0000-0003-0354-9448 (Jie Zhong)

0000-0001-5321-8941 (Hongbing Yao)

0000-0003-2613-603X (Libing Yun)

0000-0001-8965-0138 (Jianbo Li)

0009-0007-1522-7039 (Junbao Yang)

0000-0002-2192-3539 (Yan Cai)

0009-0003-9889-6465 (Hong Deng)

0000-0002-0359-4508 (Jiangwei Yan)

0000-0002-9038-2342 (Bofeng Zhu)

0009-0000-8797-9431 (Kun Zhou)

0000-0001-6414-6621 (Shengjie Nie)

0000-0001-5633-3929 (Chao Liu)

0000-0002-3673-1855 (Mengge Wang)

0000-0002-6614-5267 (Guanglin He)
