# Supplementary material for: YanHuang Paternal Genomic Resource Suggests A Weakly-differentiated Multi-source Admixture in the Formation of Han Founding Ancestral Lineages
Source: Genomics Proteomics Bioinformatics. 2025 Jun 4;23(6):qzaf049. doi: 10.1093/gpbjnl/qzaf049 (PMC13183677; doi:10.1093/gpbjnl/qzaf049)
Supplement: qzaf049_Supplementary_Data [file qzaf049_supplementary_data.zip › Supplementary material captions.docx]

# Supplementary material

**Figure S1 The geographical distribution of different haplogroups**

Geographical distribution heatmaps of O1b1a2-Page59 (**A**), O1b1a1-PK4 (**B**), O2a2b1a1-M117 (**C**), O2a2b1a2a1a-F46 (**D**), and O2a1b1a1a1a-F11 (**E**) haplogroups at the third level among Han Chinese individuals. Different colors represent haplogroup frequencies (top), whereas spatial autocorrelation analysis (bottom) indicates HotSpot (high-value spatial clustering) and ColdSpot (low-value spatial clustering) regions, with different colors representing confidence intervals. The base map was officially approved with the number GS(2023)2763.

**Figure S2 Linear correlation analysis**

The linear correlation analysis between haplogroup frequency and longitude (**A**) and latitude (**B**).

**Figure S3** **The paternal genetic structure of Han Chinese**

**A.** Heatmap of pairwise genetic distance among Han Chinese individuals. **B.** Neighbor-joining tree based on pairwise genetic distance among Han Chinese individuals, with different colors representing groupings.

**Figure S4** **The multidimensional scaling analysis**

The MDS analysis is based on the pairwise genetic distance matrix among 26 Han Chinese populations, excluding Xinjiang, Ningxia, and Hainan. MDS, multidimensional scaling.

**Figure S5 Phylogenetic tree based on the maximum parsimony method among all Han Chinese**

The different colors in the tree represent different major lineages. The different colors of the outer line present different groupings.

**Figure S6**  **Median-joining network of C2b and C2a**

**A.** Median-joining network among the Han Chinese related to C2b and its sub-lineages. **B.** Median-joining network among the Han Chinese related to C2a and its sub-lineages.

**Figure S7** **Median-joining network of N1b and N1a**

**A.** Median-joining network among the Han Chinese related to N1b and its sub-lineages. **B.** Median-joining network among the Han Chinese related to N1a and its sub-lineages.

**Figure S8** **Median-joining network of Q1a and D1a**

**A.** Median-joining network among the Han Chinese related to Q1a and its sub-lineages. **B.** Median-joining network among the Han Chinese related to D1a and its sub-lineages.

**Figure S9 Admixture graph of the Han Chinese**

Admixture graph of the Han Chinese with two admixture events obtained by model fitting using ADMIXTOOLS2. The dotted line presents admixture events. The percentage on the dotted line indicates the proportion of contributed ancestry. The number in the continuous lines indicated genetic drift.

**Figure S10 Sequencing depth**

The box plot shows the sequencing depth of 5020 samples in our study.

**Figure S11**  **Geographical distribution heatmaps among 214,307 Han Chinese individuals**

Geographical distribution heatmaps of O2a (**A**), O1a (**B**), O1b (**C**), C2a (**D**), C2b (**E**), N1a (**F**), N1b (**G**), D1a (**H**), and Q1a (**I**) haplogroups at the third level among 214,307 Han Chinese individuals. Different colors represent haplogroup frequencies. The base map was officially approved with the number GS(2023)2763.

**Table S1** **The sample information for 29 Han Chinese populations in our study**

**Table S2 The haplogroup allocation of 5020 individuals with different methods**

**Table S3 Forensic parameters for 29 Han Chinese populations based on haplotypes and haplogroups**

**Table S4 The frequency of the haplogroup within different Han Chinese populations**

**Table S5 F_ST_ matrix between different groupings of the Han Chinese**

**Table S6 Different groupings in analysis of molecular variance results**

**Table S7 Fisher's exact test for exploring the difference of major haplogroups frequency between northern China and southern China**
